# Evaluation of rice grain yield and yield components of Nona Bokra chromosome segment substitution lines with the genetic background of Koshihikari, in a saline paddy field

**DOI:** 10.1093/aobpla/plz040

**Published:** 2019-07-13

**Authors:** Shiro Mitsuya, Norifumi Murakami, Tadashi Sato, Tomohiko Kazama, Kinya Toriyama, Nicola Stephanie Skoulding, Mana Kano-Nakata, Akira Yamauchi

**Affiliations:** 1 Graduate School of Bioagricultural Sciences, Nagoya University, Furo-cho, Chikusa-ku, Nagoya, Japan; 2 Graduate School of Agricultural Science, Tohoku University, Aoba-ku, Sendai, Japan; 3 Institute for Advanced Research, Nagoya University, Furo-cho, Chikusa-ku, Nagoya, Japan

**Keywords:** Chromosome segment substitution lines, grain filling, grain weight, grain yield, rice, salt stress, yield component

## Abstract

The ability to tolerate salt differs with the growth stages of rice and thus the yield components that are determined during various growth stages, are differentially affected by salt stress. In this study, we utilized chromosome segment substitution lines (CSSLs) from Nona Bokra, a salt-tolerant *indica* landrace, with the genetic background of Koshihikari, a salt-susceptible *japonica* variety. These were screened to find superior CSSLs under long-term saline conditions that showed higher grain yield and yield components in comparison to Koshihikari. One-month-old seedlings were transplanted into a paddy field without salinity. These were allowed to establish for 1 month further, then the field was flooded, with saline water maintained at 7.41 dS m^−1^ salinity until harvest. The experiments were performed twice, once in 2015 and a targeted study in 2016. Salt tolerance of growth and reproductive stage parameters was evaluated as the Salt Effect Index (SEI) which was computed as the difference in each parameter within each line between control and saline conditions. All CSSLs and Koshihikari showed a decrease in grain yield and yield components except panicle number under salinity. SL538 showed a higher SEI for grain yield compared with Koshihikari under salinity throughout the two experiments. This was attributed to the retained grain filling and harvest index, yet the mechanism was not due to maintaining Na^+^, Cl^−^ and K^+^ homeostasis. Few other CSSLs showed greater SEI for grain weight under salinity compared with Koshihikari, which might be related to low concentration of Na^+^ in leaves and panicles. These data indicate that substitution of different Nona Bokra chromosome segments independently contributed to the maintenance of grain filling and grain weight of Koshihikari under saline conditions.

## Teaser

We evaluated grain yield and yield components of Nona Bokra chromosome segment substitution lines (CSSLs) with the genetic background of Koshihikari in a saline paddy field. SL538 showed higher grain yield and grain filling than Koshihikari under salinity, which was not attributed to Na^+^, Cl^−^ and K^+^ homeostasis in this rice genotype. SL501, 502 and 503 maintained grain weight under salinity compared with Koshihikari, which was related to low Na^+^ concentration in leaves and panicles. This study implies the independent genetic control of salt tolerance of each yield component, and this may not always be related to superior salt exclusion ability.

## Introduction

Salt stress is one of many significant abiotic stresses that limit agricultural production (Munns and Tester 2008; Shahbaz and Ashraf 2013). Rice is highly sensitive to salinity, and growth and grain yield can be seriously inhibited under these conditions (Negrão *et al.* 2011; Ismail and Horie 2017). Salt stress can occur in inland regions where soil contains high concentrations of salt and in coastal delta areas via the intrusion of sea water into the fields (Vinod *et al.* 2013). Furthermore, in coastal areas, especially the delta regions that are often affected by flooding, rice is the only crop that local farmers can grow regardless of its high salt sensitivity. To make matters worse, recent climate change has been reported to expand the salinization of paddy fields in the coastal areas in south-eastern Asia via sea water flowing back into rivers and irrigation canals. Therefore, a proposed strategy to overcome this problem is to breed salt-tolerant rice varieties to improve productivity in such salt-affected areas and to provide more choices of rice varieties to farmers.

The early seedling and reproductive stages of rice are generally more sensitive to salt in comparison to the vegetative stage. However, varieties that are salt-tolerant at the early seedling stage do not always show tolerance at the reproductive stage (Zeng *et al.* 2002; Moradi *et al.* 2003; Singh and Flowers 2010), indicating that different sets of quantitative trait loci (QTLs)/genes separately contribute to the salt tolerance at each growth stage. During the early seedling stage, maintaining low Na^+^ concentration in shoots is one of the most important traits for retaining growth and increasing the survival rate in rice (Platten *et al.* 2013; Rahman *et al.* 2016). However, despite the importance of ion exclusion (Munns and Tester 2008), there is no predominant mechanism of salinity tolerance in rice (Pires *et al.* 2015). A number of QTLs related to physiological traits have been reported (Gregorio 1997; Prasad *et al.* 1999; Flowers *et al.* 2000; Koyama *et al.* 2001; Bonilla *et al.* 2002; Lin *et al.* 2004; Lee *et al.* 2007; Mohammadi-Nejad *et al.* 2008; Kim *et al.* 2009; Sabouri *et al.* 2009; Thomson *et al.* 2010; Gimhani *et al.* 2016; Rahman *et al.* 2017) including major QTLs for salt tolerance such as *SKC1* (Lin *et al.* 2004) and *Saltol* (Bonilla *et al.* 2002; Thomson *et al.* 2010) on chromosome 1. Ren *et al.* (2005) identified the causal gene of *SKC1*, a sodium transporter *OsHKT1;5* in the *SKC1* locus and it is reported to be expressed in xylem parenchyma cells, and retrieves Na^+^ ions from xylem sap under salinity.

The mechanism by which rice grain yield decreases under salinity is thought to be highly complicated compared with that in the early seedling stage. Rice grain yield is determined by panicle number, spikelet number per panicle, grain weight and grain filling (or the percentage of ripened grains per total grains). Salt affects the grain yield by affecting several growth and physiological traits such as tillering, panicle formation, fertilization, production of photosynthate and its translocation to panicles during vegetative and reproductive stages. Maintenance of yield components has also been reported to be attributed to low Na^+^ concentration and Na^+^/K^+^ ratio in flag leaves (Hossain *et al.* 2015), floral parts (Abdullah *et al.* 2001) and panicles (Asch *et al.* 1999). High concentrations of Cl^−^ are also toxic to plants and decrease rice grain yield (Kumar and Khare 2016), whereas Na^+^ is the primary cause of ion-specific damage in many plants such as rice (Lin and Kao 2001; Tester and Davenport 2003). However, most research has focused on Na^+^, with much less attention to Cl^−^, and thus it is not clear whether Cl^−^ concentration in plants is related to the genetic variation in grain yield and yield components under salinity.

In rice, the salt sensitivity of each yield component differs and is dependent on each individual rice variety as to which component is mostly affected (Makihara *et al.* 1999; Grattan *et al.* 2002; Zeng *et al.* 2002). This indicates that different QTL/genes and physiological mechanisms may regulate each yield component under salinity. Although recent QTL mapping studies have reported a variety of QTL using F_2_ progeny between salt-sensitive and -tolerant varieties at the reproductive stage (Mohammadi *et al.* 2013; Hossain *et al.* 2015), studies on QTL mapping for grain yield and yield components of rice under salinity are still limited compared with those for salt tolerance QTL at the seedling stage. It also implies the possibility to improve rice productivity under salinity by pyramiding the QTLs for retaining each yield component, although this has not yet been proven genetically.

We hypothesized that a potential number of different chromosome segments improves each yield component independently and overall grain yield in rice under long-term salinity. To test this hypothesis, we evaluated the grain yield and yield components of Koshihikari (a salt-sensitive Japanese leading *japonica* variety) and Nona Bokra (a salt-tolerant *indica* landrace) chromosome segment substitution lines (CSSLs) with the genetic background of Koshihikari under long-term salinity. Each Nona Bokra CSSLs (44 lines in total) contains only small parts of the Nona Bokra chromosome, of which the percentage of substituted segment(s) ranges between 2.1 and 11.7 % (Takai *et al.* 2007). Each chromosome was covered by three or four lines carrying overlapping segments, except for a small region at the distal end of the short arm on chromosome 1, which was not covered (Takai *et al.* 2007). In addition, genetically nearly homozygous CSSLs enable us to eliminate the genetic variation among CSSLs under non-saline conditions. For this purpose, we used a Salt Effect Index (SEI) for the evaluation of grain yield, yield components and growth parameters (above-ground biomass and harvest index) under salinity by computing the difference in each parameter within each line between control and saline conditions. This equation is adapted from ‘Root Plasticity’ studies (Sandhu *et al.* 2016; Nguyen *et al.* 2018; Suralta *et al.* 2018), where the authors used it to calculate the response of root phenotypic parameters to a stress treatment compared to the control conditions. This SEI is effective when comparing responses of various parameters between control and treatment conditions among genotypes in a split plot design experiment with treatments arranged as main plots, whereas the parameters show genotypic variation under control conditions. By comparing the SEI of grain yield, yield components and growth parameters (above-ground biomass and harvest index) between Koshihikari and Nona Bokra CSSLs, we screened for genotypes that maintained grain yield under salinity and determined the causal yield components and growth parameters. Then we determined whether the maintenance of grain yield and yield components in the salt-tolerant CSSLs under salinity was related to the concentrations of Na^+^, Cl^−^, K^+^ or Na^+^/K^+^ ratio in plants.

## Materials and Methods

### Plant materials

The seeds of Nona Bokra CSSLs (SL501–SL544) derived from Nona Bokra and Koshihikari cross (44 lines in total; Takai *et al.* 2007) and the recurrent parent Koshihikari were received from the Rice Genome Resource Center, Tsukuba, Japan. The graphical genotypes of Nona Bokra CSSLs have been published in Takai *et al.* (2007) and the genotype data were obtained from the website of the Rice Genome Resource Center (2007). They were grown in 2012 for seed increase and the collected seeds were used for this study. All 44 of Nona Bokra CSSLs and the recurrent parent Koshihikari were used for the experiment in 2015, except SL503 and SL516 that grew poorly in the nursery bed and were not transplanted to the fields. In 2016, selected lines (SL501, 503, 507, 511, 522, 524, 534, 538, 539, 541, 542) and Koshihikari were used.

### Salt treatment

The experiment was conducted twice, in 2015 and 2016, at the experimental farm station, Graduate School of Life Science, Tohoku University at Kashimadai (37°28′N, 141°06′E) in Miyagi Prefecture, Japan. Nitrogen, phosphorus and potassium fertilizers were applied as basal at the rates of 30 kg ha^−1^ each in both years. Thirty- and 35-day-old seedlings in 2015 and 2016, respectively, were transplanted at 11.1 hills per m^2^ (1 plant per hill) at a spacing of 30 cm between hills as well as rows on 28 May in 2015 and 24 May in 2016. The experiment was arranged in a split plot design in a randomized complete block design with salt treatments as main plots while Koshihikari and CSSLs were arranged as subplots. The 42 CSSLs and Koshihikari in 2015, and 10 CSSLs and Koshihikari in 2016, were transplanted in single row plots (5 plants per plot) with three replications. These were allowed to establish for ~1 month, then the field was flooded with saline water maintained at an electrical conductivity (EC) of 7.41 dS m^−1^. This was measured by a portable salt meter (APAL-ES1, AS ONE cooperation, Osaka, Japan) using well water containing salt (major ions were Na^+^ and Cl^−^) and mixed with irrigation water containing less salt, until harvest. The control field was irrigated with irrigation water where salt concentration was undetectable (<0.21 dS m^−1^). The salt treatment initiation date was 23 June in 2015 and 21 June in 2016 when plants were 56 and 63 days old, respectively, and were at the active-tillering stage.

**Figure 1.**
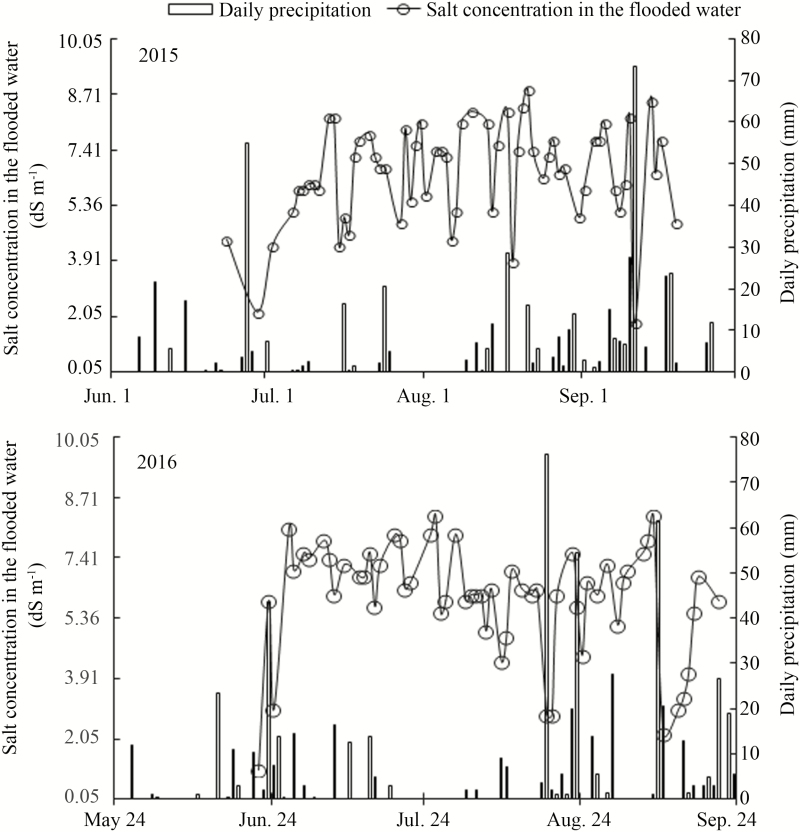
Changes in salt concentration in the flood water of the saline well water-irrigated paddy field in 2015 and 2016. Daily rainfall during the experiments is also shown. The salt concentration in the flood water shown was measured before adjusting it to 7.41 dS m^−1^. Salt concentration in the flooded water of the control field was under the detection limit of the equipment (<0.21 dS m^−1^). Transplanting was done on 28 May in 2015 and 24 May in 2016. Salt treatment was initiated on 24 June in 2015 and 21 June in 2016 when plants were actively tillering, till harvest on 25 September in 2015 and 23 September in 2016. Harvest for measurement of Na^+^, K^+^ and Cl^−^ concentrations was performed on 1 September in 2016.

After filtering using a 0.22-μm membrane filter, the ion concentration in the saline well water and irrigation water was measured with inductively coupled plasma atomic emission spectrometry (ICP-AES) (IRIS ICAP, Nippon Jarrell Ash, Kyoto, Japan) and ion chromatography for Cl^−^ (Shim-Pack IC-A3, Shimadzu, Kyoto, Japan) **[see** **Supporting Information—Table S1****]**. The salt concentration in the flood water shown in Fig. 1 was measured daily using the above-mentioned portable salt meter before adjusting the salt concentration and expressed as the mean of four different measurements. Additionally, apparent soil electrical conductivity (EC_a_) in the top soil was measured at ~5 cm below the soil surface before treatment and 1 month after the initiation of the treatment using a 5TE EC sensor (METER Group, Pullman, WA, USA) and expressed as the mean of six different measurements (Table 1). To make the regression curve between the EC of the saturated soil-paste extract (EC_e_) and EC_a_, soil was randomly sampled and used for the measurement of EC of 1:5 soil to water extract (EC_1:5_), which was then converted to EC_e_ using the following equation (Dobermann and Fairhurst 2000):

**Table 1.** Apparent soil electrical conductivity in the experimental fields. Apparent soil electrical conductivity (EC_a_) at ~5 cm below the soil surface was measured before the initiation of treatment and 1 month after the initiation of the treatment. The values are the mean average of six different measurements (two measurements from each replicate) and the standard deviation. The regression curve between the electrical conductivity of the saturated soil-paste extract (EC_e_) and EC_a_ was expressed as EC_e_ = 2.2392 EC_a_ − 40.586 (*r* = 0.996).

		Apparent soil electrical conductivity (dS m^−1^)	
Experimental year	Treatment	Before treatment	Treatment
2015	Control	0.21 ± 0.015	0.44 ± 0.067
	Salinity	0.68 ± 0.060	2.67 ± 0.21
2016	Control	0.23 ± 0.043	0.31 ± 0.053
	Salinity	1.39 ± 0.19	3.25 ± 0.21

$$\begin{array}{l} EC_{e}=6.4\times EC_{1:5} \end{array}$$

Climatic data were obtained from automated meteorological data acquisition system (AMeDAS) in Kashimadai station (38°27′N, 141°05′E) **[see** **Supporting Information—Table S2****and** **Fig. S1****]**.

### Measurement of biomass, yield and yield components and computation of SEI

The harvested plants at maturity were air-dried in a vinyl greenhouse for 1 month and used for the measurement of panicle weight. Two average-sized hills per plot were then selected to determine each parameter. Panicles were cut at the panicle base and weighed. For grain yield measurement, after all spikelets were separated from all panicles, we separated filled grains from poorly filled and sterile grains using tap water. Then the unfilled or sterile grains were further separated into poorly filled grain and sterile grains using 70 % (v/v) ethanol according to Kobata *et al.* (2010). The filled grains with the husk not removed were weighed and the moisture content was measured using a grain moisture tester (Riceter-f, Kett, Tokyo, Japan) and the grain yield was expressed as 15 % moisture content unhulled grain weight. Grain filling (%) was calculated by dividing the number of filled grains by the total spikelet number. The remained above-ground parts were dried at 70 °C for >72 h and used for the measurement of above-ground biomass. Harvest index (%) was calculated by dividing grain yield by above-ground biomass.

Salt Effect Index was calculated using each replicate from the salt treatment and mean values from the control treatment as follows:

$$\begin{array}{l} Salt\;Effect\;Index\;(SEI)=\frac{X_{salt}-{\bar{X}}_{control}}{{\bar{X}}_{control}} \end{array}$$

where *X*_salt_ is the individual data of each trait under salt treatment and ${\bar{X}}_{control}$ indicates the mean value of the trait among three replicates under control conditions.

In 2015, we evaluated the SEI of panicle weight of 42 CSSLs and Koshihikari. The top six CSSLs (SL501, 502, 534, 538, 541, 542) with the highest SEI of panicle weight, the bottom four CSSLs (SL507, 511, 522, 539) with the lowest, and Koshihikari, were evaluated for grain yield and yield components. In 2016, the same CSSLs and Koshihikari were used for analyses except that SL502. SL503 that harbours *SKC1* QTL (Takai *et al.* 2007) was also used in the second year to see the effect of the QTL introgression on the grain yield and yield components under salinity.

### Na^+^, K^+^ and Cl^−^ measurement

Shoot parts harvested at about 1 month after heading were separated into leaf blades, leaf sheaths and developing panicles, dried in an oven set at 70 °C for >72 h, finely powdered, and used for ion extraction with 0.1 M acetic acid as described in Platten *et al.* (2013). Three average-sized hills per plot were selected and used for measurement. Whole leaf blades, sheaths and panicles were used. The concentrations of cation (Na^+^, K^+^) and Cl^−^ were measured using atomic absorption spectrometry (iCE 3000, Thermo Scientific, Franklin, MA, USA) and ion chromatography, respectively.

### Statistics

Statistical analyses were carried out using the R statistical package (R Core Team 2013). Data sets from each year were analysed separately. One-way analysis of variance (ANOVA) was performed to assess the effects of the line, and two-way ANOVA for the effect of the line, the treatment and their interaction. Line and treatment were fixed independent variables. The means of each parameter were compared between Koshihikari and each CSSL using Dunnett’s test at 0.05, 0.01 and 0.001 probability levels. Pearson’s coefficient of correlation was used for correlation studies described in this study.

## Results

### Changes in the salt concentration in flood water

In this study, salt treatment was performed by irrigating with a mix of saline well water and irrigation water to maintain the salt concentration in the flood water at 7.41 dS m^−1^; however, the salt concentration fluctuated between 3.91 and 8.71 dS m^−1^ in most days due to rainfall and/or evaporation of the flood water (Fig. 1). The salt concentration under control conditions is not shown in Fig. 1 since it was under the threshold level that the equipment can detect. After the initiation of salt treatment, the apparent soil EC (EC_a_) reached to 2.67 dS m^−1^ in 2015 and 3.24 dS m^−1^ in 2016 while the EC_a_ remained to be low under control treatment (0.44 dS m^−1^ in 2015 and 0.30 dS m^−1^ in 2016) (Table 1). Prior to the initiation of salt treatment in the salinity plot, the EC_a_ was higher than in the control plot. This was most likely due to previous years salinity experiments leaving residual salt in the soil.

The major ions contained in the well water were Cl^−^ (8211.47 ppm) and Na^+^ (2344.26 ppm), and they accounted for >90 % of the total amount of measured ions **[see** **Supporting Information—Table S1****]**. The saline well water also contained higher concentrations of B, Ca, Fe, K, Mg, Mn, Si and P than control irrigation water.

### Grain yield and yield components

**Figure 2.**
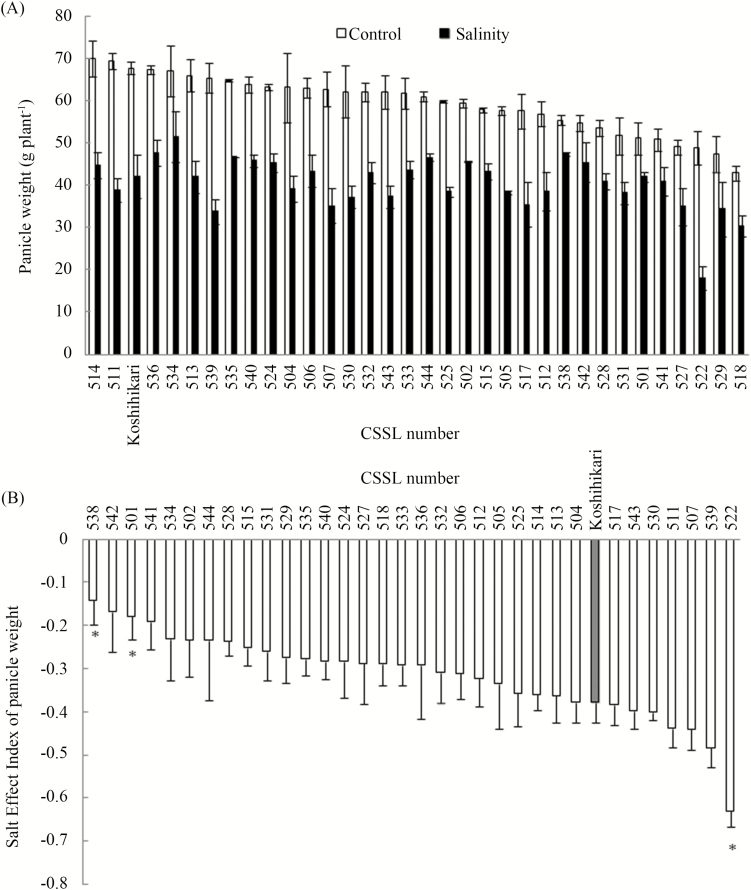
Panicle weight of Koshihikari and Nona Bokra CSSLs grown in control and saline fields (A) and its SEI (B) in 2015. Values are mean ± SEM (*n* = 3). * indicates a significant difference from Koshihikari at the 0.05 probability level. Within 44 Nona Bokra CSSLs, SL503 and 516 grew poorly in the nursery bed and were not transplanted to the fields in 2015. SL508, 509, 510, 519, 520, 521, 523, 526, 537 produced no panicle or sterile panicles thus not included.

The 2015 experiment was conducted to identify and target the Nona Bokra CSSLs that showed a different response to salinity compared with Koshihikari. We firstly measured the panicle weight of Koshihikari and all the CSSLs, except SL503 and SL516 that grew poorly in the nursery bed and were not transplanted to the fields in 2015. Within the 42 CSSLs, eight late-heading lines (SL509, 510, 519, 520, 521, 523, 526 and 537) showed sterile panicles under both control and saline conditions. Another late-heading line SL508 did not produce any panicle under salinity. Except these CSSLs, the panicle weight of Koshihikari and other CSSLs ranged from 42.86 to 69.96 g per plant under control conditions (Fig. 2A). Salt treatment decreased panicle weight in the range of 18.10 to 51.50 g per plant. The SEI of panicle weight of Koshihikari was −0.378. The Nona Bokra CSSLs showed a wide range of SEI of panicle weight (−0.141 to −0.629). SL538 and 501 showed a significantly higher SEI than Koshihikari, whereas that of SL522 was significantly lower (Fig. 2B).

Table 2 shows the SEI of grain yield, yield components, above-ground biomass and harvest index of Koshihikari and selected CSSLs under control and saline conditions. We used the top six CSSLs with the highest SEI of panicle weight (SL501, 502, 534, 538, 541, 542), the bottom four CSSLs (SL507, 511, 522, 539) with the lowest and Koshihikari for comparison. SL503 in 2016 that harbour *SKC1* QTL (Takai *et al.* 2007) was also used to see the effect of the QTL introgression on the grain yield and yield components under salinity. Salt treatment decreased the grain yield of Koshihikari and the SEI was −0.621 in 2015 and −0.902 in 2016. In Koshihikari, all yield components except panicle number decreased under salinity, with grain filling reduced most, followed by spikelet number per panicle and then grain weight (Table 2; also **see** **Supporting Information—Table S3**).

**Table 2.** Salt Effect Index of Koshihikari and Nona Bokra CSSLs. In 2015, Koshihikari and 10 Nona Bokra CSSLs (the top six CSSLs with the highest SEI of panicle weight, the bottom four CSSLs with the lowest) were evaluated. In 2016, the same CSSLs and Koshihikari were used for analyses except that SL502 was replaced with SL503. Salt Effect Index was calculated using each replicate from the salt treatment and mean values from the control treatment as follows: $Salt\;Ef\;fect\;Index\;(SEI)=(X_{salt}-{\bar{X}}_{control})/{\bar{X}}_{control}$, where *X*_salt_ is the individual data of each trait under salt treatment and ${\bar{X}}_{control}$ indicates the mean value of the trait among three replicates under control conditions. *, ** and *** indicate a significant difference from Koshihikari at *P* < 0.05, 0.01 and 0.001, respectively (Dunnett’s test). In analysis of variance, *, ** and *** indicate at the 5, 1 and 0.1 % level significance, respectively. n.s., not significant.

CSSL no.	Grain yield	Panicle number	Spikelet number per panicle	Grain weight	Grain filling	Above-ground biomass	Harvest index
2015 experimental year							
Koshihikari	−0.621	0.092	−0.284	−0.157	−0.415	−0.315	−0.385
501	−0.401	0.180	−0.208	−0.046**	−0.335	−0.151	−0.297
502	−0.638	0.303	−0.320	−0.056**	−0.564	−0.147	−0.574
507	−0.702	0.239	−0.247	−0.246*	−0.584	−0.330	−0.565
511	−0.580	0.210	−0.389	−0.156	−0.316	−0.344	−0.357
522	−0.742	0.246	−0.518*	−0.203	−0.466	−0.411	−0.566
534	−0.536	0.258	−0.301	−0.185	−0.350	−0.138	−0.377
538	−0.126***	0.482*	−0.249	−0.152	−0.066*	−0.176	−0.005
539	−0.800	0.547**	−0.496*	−0.221	−0.677	−0.332	−0.708
541	−0.288*	0.341	−0.251	−0.134	−0.184	−0.155	−0.163
542	−0.307*	0.206	−0.132	−0.145	−0.242	−0.174	−0.172
2016 experimental year							
Koshihikari	−0.902	0.110	−0.395	−0.204	−0.828	−0.533	−0.799
501	−0.930	0.094	−0.411	−0.039***	−0.892	−0.480	−0.877
503	−0.823	0.028	−0.423	−0.099**	−0.689	−0.500	−0.669
507	−0.845	−0.043	−0.312	−0.196	−0.711	−0.545	−0.681
511	−0.916	0.274	−0.437	−0.260	−0.851	−0.498	−0.841
522	−0.765	−0.050	−0.425	−0.200	−0.467	−0.576	−0.453
534	−0.787	0.066	−0.317	−0.147	−0.663	−0.430	−0.659
538	−0.584*	0.221	−0.339	−0.208	−0.291**	−0.436	−0.256**
539	−0.896	0.013	−0.457	−0.211	−0.757	−0.614	−0.744
541	−0.700	0.173	−0.427	−0.213	−0.430*	−0.511	−0.387
542	−0.676	0.143	−0.297	−0.218	−0.516	−0.399	−0.491
ANOVA							
2015	***	n.s.	***	***	***	n.s.	***
2016	**	**	*	***	**	n.s.	***

Salinity reduced grain yield and all the yield components except panicle number of all the selected CSSLs (Table 2; also **see** **Supporting Information—Table S3**). Regarding grain weight, the SEI of grain yield in SL538 was the highest among the selected lines and significantly higher than that in Koshihikari in both years (Table 2). SL541 and 542 also showed higher SEI of grain yield than Koshihikari in 2015.

The yield components, specifically panicle number, grain weight and grain filling showed greater SEI in a number of CSSLs compared with Koshihikari (Table 2). The SEI of panicle number was higher in SL538 and 539 than in Koshihikari in 2015 but they were comparable in 2016. The SEI of spikelet number per panicle was lower in SL522 and 539 than in Koshihikari in 2015. Regarding grain weight, SL501 that harbours Nona Bokra *SKC1* QTL showed a higher SEI than Koshihikari in both 2015 and 2016. SL502 and 503 that harbour Nona Bokra *SKC1* QTL also showed higher SEI of grain weight than Koshihikari in the experimental year they were used. SL507 also showed a higher SEI of grain weight in 2015 but not in 2016. The SEI of grain filling was higher in SL538 in both years and in SL541 in 2016 than in Koshihikari.

Regarding above-ground biomass and harvest index, the SEI of harvest index was higher in SL538 than in Koshihikari in both years (Table 2). SL541 also showed higher SEI for harvest index than Koshihikari in 2016. The SEI of above-ground biomass was comparable among all lines.

ANOVA results revealed that the line had significant effect on SEI of grain yield, yield components and agronomic parameters except panicle number in 2015 and above-ground biomass in 2015 and 2016 (Table 2).

### Na^+^, K^+^ and Cl^−^ concentrations and Na^+^/K^+^ ratio under salinity

**Figure 3.**
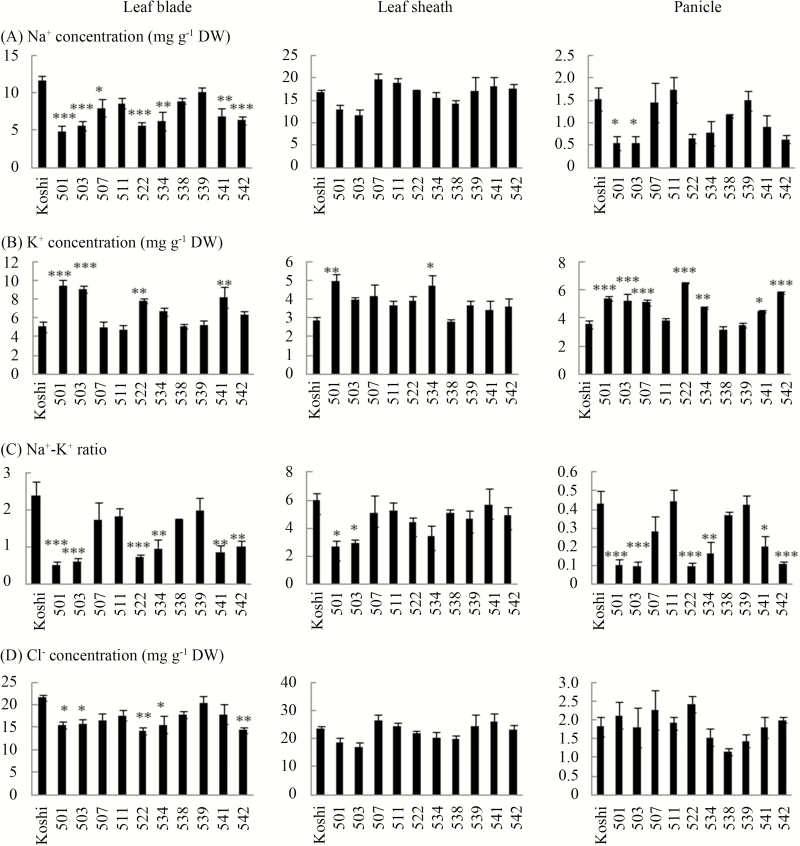
Na^+^, K^+^ and Cl^−^ concentrations and Na^+^-K^+^ ratio in the whole leaf blades, leaf sheaths and panicles of Koshihikari and Nona Bokra CSSLs under saline conditions. The samples were harvested about 2 weeks after heading in 2016. X-axis indicates CSSL numbers or Koshihikari. Three average-sized hills per plot were used for measurement. Values are mean ± SEM (*n* = 3). *, ** and *** indicate a significant difference from Koshihikari at 0.05, 0.01 and 0.001, respectively (Dunnett’s test). Koshi, Koshihikari.

To determine whether the concentration of Na^+^, K^+^ and Cl^−^ is related to variation in grain yield under salinity, we determined the Na^+^, K^+^ and Cl^−^ concentrations and Na^+^/K^+^ ratio in leaf blades, leaf sheaths and panicles that were harvested at 2 weeks after heading. Regarding Na^+^, most of the CSSLs except SL511, 538, 539 showed a lower Na^+^ concentration than Koshihikari in leaf blades (Fig. 3). In panicles, SL501 and 503 showed a significantly lower Na^+^ concentration than Koshihikari.

SL501, 503, 522 and 541 showed higher K^+^ concentration in leaf blades compared with Koshihikari (Fig. 3). SL501 and 534 showed higher K^+^ concentration in leaf sheath than Koshihikari. In panicles, most of the CSSLs except SL511, 538 and 539 showed higher K^+^ concentration than that in Koshihikari. SL501 especially showed significantly higher K^+^ concentration in all organs than Koshihikari.

In leaf blades and panicles, Na^+^ concentration was negatively correlated with K^+^ concentration (data not shown). SL501, 503, 522, 534, 541 and 542 showed a significantly lower Na^+^/K^+^ ratio than Koshihikari in leaf blades and panicles (Fig. 3). SL501 and 503 showed lower Na^+^/K^+^ ratio than Koshihikari in leaf sheaths.

SL501, 503, 522, 524, 534 and 542 showed significantly lower Cl^−^ in comparison to Koshihikari in leaf blades (Fig. 3).

To see whether the variation in maintaining grain yield, yield components and growth parameters (above-ground biomass and harvest index) under salinity among Nona Bokra CSSLs is related to the variation of salt exclusion ability, correlation analysis was performed between the concentrations of Na^+^, K^+^ and Cl^−^ and Na^+^/K^+^ ratio and the SEI of growth and reproductive stage parameters under salinity. Among the determined parameters, only SEI of grain weight showed significant correlation (Table 3). The SEI of grain weight showed a significantly negative correlation with Na^+^ concentration in all the determined organs (Table 3). K^+^ concentration and Na^+^/K^+^ ratio also showed a highly positive and negative correlation, respectively, with the SEI of grain weight in all the determined organs. Cl^−^ showed a significantly negative correlation with the SEI of grain weight only in leaf sheath. These results indicate that maintaining low concentrations of Na^+^, Cl^−^, low Na^+^/K^+^ ratio and high K^+^ concentration contributed to maintaining grain weight under saline conditions.

**Table 3.** Pearson correlation coefficients between Na^+^, K^+^ and Cl^−^ concentrations and Na^+^-K^+^ ratio in whole leaf blades, leaf sheath and panicles under salinity and SEI for grain yield, yield components and growth parameters of Koshihikari and 10 Nona Bokra CSSLs. Data in 2016 were used. Mean values of three replicates of each genotype were used for calculation. **P < 0.01, *P < 0.5.

Organ	Ion species	Grain yield	Panicle number	Spikelet number per panicle	Grain filling	Grain weight	Above-ground biomass	Harvest index
Leaf blade	Na^+^	−0.179	0.225	−0.105	−0.166	−0.579*	−0.341	−0.147
	K^+^	0.045	−0.232	−0.259	0.088	0.651*	0.064	0.078
	Cl^−^	−0.241	0.226	−0.317	−0.212	−0.372	−0.396	−0.193
	Na^+^-K^+^ ratio	−0.229	0.220	0.007	−0.239	−0.571	−0.278	−0.220
Leaf sheath	Na^+^	−0.086	0.060	0.098	−0.040	−0.707*	−0.250	−0.025
	K^+^	−0.387	−0.428	0.077	−0.404	0.652*	0.066	−0.429
	Cl^−^	−0.098	0.044	0.005	−0.035	−0.678*	−0.358	−0.011
	Na^+^-K^+^ ratio	0.191	0.352	0.053	0.217	−0.813**	−0.153	0.247
Panicle	Na^+^	−0.354	0.262	−0.147	−0.327	−0.613*	−0.384	−0.310
	K^+^	0.053	−0.521	0.169	0.087	0.357	0.079	0.073
	Cl^−^	−0.357	−0.414	−0.044	−0.287	0.150	−0.229	−0.282
	Na^+^-K^+^ ratio	−0.252	0.379	−0.210	−0.234	−0.587*	−0.324	−0.220

## Discussion

In this study, we used a salt-sensitive Japanese *japonica* rice variety Koshihikari and a salt-tolerant *indica* rice landrace, Nona Bokra CSSLs with the genetic background of Koshihikari (Takai *et al.* 2007) for identifying Nona Bokra chromosome segments that contribute to the retained grain yield and yield components under salinity. Although each CSSL harbours only 2.1 to 11.7 % of Nona Bokra chromosome segments (Takai *et al.* 2007), they showed a wide range of grain yield and yield component variation **[see** **Supporting Information—Table S3****]**. Therefore, we used SEI which was calculated by comparing each parameter (grain yield, yield components, above-ground biomass and harvest index) between control and saline conditions.

In both experiments, SL538 showed greater grain yield and SEI than Koshihikari under salinity, whereas their grain yields were statistically not different under control conditions (Table 2; also **see** **Supporting Information—Table S3**). Also, SL538 showed greater SEI of grain filling and harvest index than Koshihikari but for the other yield components, SEI was comparable. This result indicates that the introgressed segments of Nona Bokra chromosomes in SL538 specifically function in retaining grain filling and harvest index under salinity, and thus the grain yield was maintained under salinity. Grain filling and harvest index are determined by the percentage of fertilization and remobilization of pre-stored and concurrently produced photosynthates from vegetative tissues to panicles. Also, grain filling is directly linked to panicle fertility and directly affects harvest index. In this study, salinity decreased fertilization percentage in both Koshihikari and SL538; however, the degree of reduction was not significantly different (data not shown). Therefore, this indicated that the greater ability of SL538 to retain grain filling and harvest index under salinity might be related to remobilization of pre-stored and concurrently produced photosynthates from vegetative tissues to panicles. Interestingly, we found that the greater grain yield, grain filling and harvest index of SL538 under salinity was not attributed to maintained Na^+^, K^+^ and Cl^−^ homeostasis in leaf blades, leaf sheaths and panicles (Table 2; Fig. 3). Castillo *et al.* (2007) also reported that the decrease in harvest index under salinity was not attributed to the ionic component of salt stress. Further study is needed to elucidate the mechanism by which SL538 maintained grain filling and grain yield under long-term salinity.

SL538 harbours Nona Bokra chromosome 11 and small parts of chromosome 12, whereas SKC1, a major QTL for salt tolerance (Lin *et al.* 2004) from Nona Bokra on chromosome 1, is absent in SL538 **[see** **Supporting Information—Fig. S2****]** (Takai *et al.* 2007). SL541 harbouring a part of Nona Bokra chromosome 11 **[see** **Supporting Information—Fig. S2****]** also showed higher SEI for grain filling and harvest index under salinity compared with Koshihikari in 2016 (Table 2), which indicates salt tolerance-related QTLs in the overlapped part of Nona Bokra chromosome 11 between SL538 and SL541 at the simple sequence repeat (SSR) markers RM6623 and RM5926 **[see** **Supporting Information—Fig. S2****]** (Takai *et al.* 2007). So far, salt tolerance-related QTLs on rice chromosome 11 have been reported such as that for maintaining grain yield (Tiwari *et al.* 2016) and that for leaf bronzing (Takehisa *et al.* 2006) under saline conditions. There is no salt tolerance-related QTL reported so far between the SSR markers RM6623 and RM5926, though *OsNHX2* is localized in the region within salt tolerance-related genes (Negrão *et al.* 2011). Narrowing down the causal QTLs using SL538 for higher grain yield under long-term saline conditions enables efficient salt tolerance breeding, such as pyramiding with other salt tolerance-related QTLs like *SKC1*.

We found that SL501, 502 and 503, which share a part of Nona Bokra chromosome 1 **[see** **Supporting Information—Fig. S2****]**, showed greater ability to maintain grain weight under salinity (Table 2). Grain weight is determined by the size of grain during panicle development and remobilization of pre-stored and concurrently produced photosynthates from leaf sheaths and stems to panicles during grain filling. The growth stages determining those physiological mechanisms seem somehow overlapped with those for grain filling; however, the CSSLs showing greater SEI in grain weight and grain filling did not overlap. It suggests that the maintenance of grain filling and grain weight under salinity may be controlled by different QTLs and physiological mechanisms. The grain weight SEI had a significantly negative correlation with Na^+^ concentration in leaf blades, leaf sheaths and panicles (Table 3). In addition, SL501 and 503 showed significantly lower Na^+^-K^+^ ratio in leaf blades and panicles than Koshihikari (Fig. 3). Therefore, the maintenance of grain weight under salinity may potentially be attributed to lower Na^+^ in panicles, leaf blades and leaf sheaths. It was indicated that lower Cl^−^ concentration in leaf sheath may also be related to the maintenance of grain weight (Table 3). Since Cl^−^ has been reported to have little or no toxicity to rice growth compared with Na^+^ (Lin and Kao 2001; Tester and Davenport 2003), the result implied the importance of lowering Cl^−^ concentration in those organs. On the other hand, Cl^−^ concentration in leaf sheath was highly and positively correlated with Na^+^ concentration in leaf sheath (data not shown). Therefore, further study is necessary to validate the relationship between Cl^−^ concentration and grain weight under salinity.

Interestingly, some CSSLs showing greater SEI for grain weight (SL501, 502, 503) harbour the overlapped segments of Nona Bokra chromosome 1 between the SSR markers RM1032 and RM7266 **[see** **Supporting Information—Fig. S2****]** (Takai *et al.* 2007) where several QTLs such as *SKC1* (Lin *et al.* 2004; Ren *et al.* 2005), *Saltol* (Gregorio 1997; Bonilla *et al.* 2002; Thomson *et al.* 2010), *qSKC-1* and *qSNC-1* (Zheng *et al.* 2015; Jing *et al.* 2017) are also localized and control the concentrations of Na^+^ and K^+^ and the Na^+^/K^+^ ratio in shoots. This implies that the introgression of these QTL may confer the ability to retain grain weight under salinity. Further research is needed to determine the causal QTL in the Nona Bokra chromosome 1 for maintaining grain weight under salinity. It also indicates the importance of the identification of QTL for maintaining each yield component and the possibility of QTL pyramiding for increasing grain yield under salinity.

## Conclusions

We found that SL538 showed greater grain yield than Koshihikari under salinity, which was attributed to higher grain filling. Also, the greater grain filling in SL538 under salinity might be related to a higher harvest index. The salt tolerance mechanisms in SL538 were not attributed to Na^+^, Cl^−^ and K^+^ homeostasis in this rice genotype. SL501, 502 and 503 maintained grain weight under salinity compared with Koshihikari, which was related to low Na^+^ concentration in leaves and panicles. This study implies the independent genetic control of salt tolerance of each yield component, and this may not be always related to superior salt exclusion ability.

## Sources of Funding

This work was supported by a Grant-in-Aid for Scientific Research from the Japan Society for the Promotion of Science (nos. 25292012 and 19H02942) and partially supported by the Japan Science and Technology Agency (JST)/Japan International Cooperation Agency (JICA), the Science and Technology Research Partnership for Sustainable Development (SATREPS).

## Contributions by the Authors

S.M., N.M. and T.S. designed and performed research. S.M. and N.M. collected and analysed data. S.M. wrote the paper. S.M., T.S., T.K., K.T., N.S.S., M.K.-N. and A.Y. discussed and revised the manuscript. All authors read and approved the final manuscript.

## Conflict of interest

None declared.

## Supplementary Material

plz040_suppl_Supplementary_Figure_and_Tables

plz040_suppl_Supplementary_Data

## Supporting Information

The following additional information is available in the online version of this article—

**Figure S1.** Daily maximum, minimum and average air temperature during the experiments.

**Figure S2.** Substitution positions with Nona Bokra chromosomes in SL501, 502, 503, 538 and 541.

**Table S1.** The concentrations of ions in the irrigation water and saline well water.

**Table S2.** Average monthly temperature, rainfall and duration of sunshine during the growth period (June to September) in 2015 and 2016.

**Table S3.** Grain yield, yield components and agronomic parameters of Koshihikari and Nona Bokra chromosome segment substitution lines (CSSLs) under control and saline conditions in 2015 and 2016.
