# Supplementary material for: Evaluation of rice grain yield and yield components of Nona Bokra chromosome segment substitution lines with the genetic background of Koshihikari, in a saline paddy field
Source: AoB Plants. 2019 Jul 13;11(5):plz040. doi: 10.1093/aobpla/plz040 (PMC6790112; doi:10.1093/aobpla/plz040)
Supplement: plz040_suppl_Supplementary_Figure_and_Tables [file plz040_suppl_supplementary_figure_and_tables.pptx]

## Slide 1
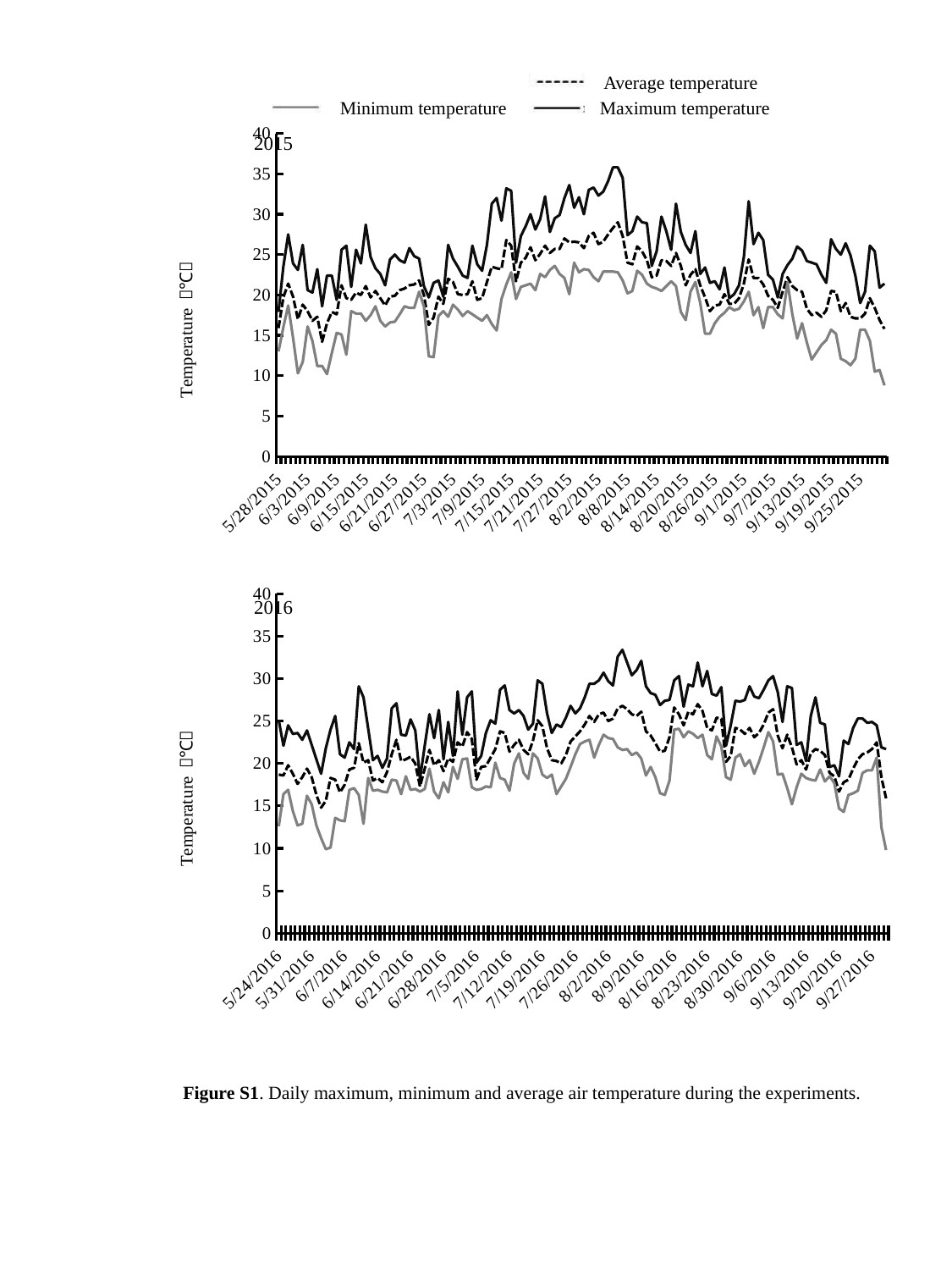

Average temperature
Minimum temperature
Maximum temperature
### Chart
| Category | 最高気温 | 平均気温 | |
|---|---|---|---|
| 42152 | 17.9 | 15.9 | 13.0 |
| 42153 | 23.5 | 19.7 | 16.0 |
| 42154 | 27.5 | 21.4 | 18.7 |
| 42155 | 23.9 | 19.8 | 14.7 |
| 42156 | 23.1 | 17.0 | 10.3 |
| 42157 | 26.2 | 18.8 | 11.7 |
| 42158 | 20.6 | 18.0 | 16.1 |
| 42159 | 20.3 | 16.8 | 14.3 |
| 42160 | 23.2 | 17.3 | 11.2 |
| 42161 | 18.6 | 14.2 | 11.2 |
| 42162 | 22.4 | 16.5 | 10.200000000000001 |
| 42163 | 22.4 | 17.9 | 12.8 |
| 42164 | 19.4 | 17.6 | 15.3 |
| 42165 | 25.6 | 21.2 | 15.1 |
| 42166 | 26.1 | 19.6 | 12.6 |
| 42167 | 21.0 | 19.4 | 18.0 |
| 42168 | 25.6 | 20.3 | 17.7 |
| 42169 | 23.9 | 20.0 | 17.7 |
| 42170 | 28.7 | 21.1 | 16.8 |
| 42171 | 24.7 | 19.7 | 17.5 |
| 42172 | 23.3 | 20.5 | 18.6 |
| 42173 | 22.6 | 19.6 | 16.8 |
| 42174 | 21.2 | 18.7 | 16.1 |
| 42175 | 24.4 | 19.8 | 16.6 |
| 42176 | 25.0 | 19.9 | 16.7 |
| 42177 | 24.3 | 20.6 | 17.6 |
| 42178 | 24.0 | 20.8 | 18.6 |
| 42179 | 25.8 | 21.2 | 18.4 |
| 42180 | 24.8 | 21.3 | 18.4 |
| 42181 | 24.5 | 21.8 | 20.4 |
| 42182 | 21.1 | 20.0 | 18.6 |
| 42183 | 19.7 | 16.3 | 12.4 |
| 42184 | 21.5 | 17.2 | 12.3 |
| 42185 | 21.8 | 19.8 | 17.4 |
| 42186 | 19.8 | 18.9 | 18.0 |
| 42187 | 26.2 | 22.0 | 17.3 |
| 42188 | 24.5 | 21.6 | 18.8 |
| 42189 | 23.5 | 20.1 | 18.2 |
| 42190 | 22.4 | 20.0 | 17.4 |
| 42191 | 22.1 | 20.1 | 18.0 |
| 42192 | 26.1 | 21.7 | 17.6 |
| 42193 | 23.8 | 19.4 | 17.2 |
| 42194 | 23.0 | 19.6 | 16.8 |
| 42195 | 26.2 | 21.6 | 17.5 |
| 42196 | 31.3 | 23.5 | 16.4 |
| 42197 | 32.0 | 23.3 | 15.6 |
| 42198 | 29.2 | 23.2 | 19.5 |
| 42199 | 33.2 | 26.8 | 21.3 |
| 42200 | 32.9 | 26.1 | 22.8 |
| 42201 | 24.0 | 21.7 | 19.5 |
| 42202 | 27.3 | 24.0 | 21.0 |
| 42203 | 28.5 | 24.6 | 21.2 |
| 42204 | 30.0 | 25.9 | 21.4 |
| 42205 | 28.1 | 24.3 | 20.6 |
| 42206 | 29.4 | 25.1 | 22.6 |
| 42207 | 32.2 | 26.1 | 22.2 |
| 42208 | 27.8 | 25.2 | 23.1 |
| 42209 | 29.5 | 25.7 | 23.6 |
| 42210 | 29.9 | 25.7 | 22.6 |
| 42211 | 32.0 | 27.0 | 22.1 |
| 42212 | 33.6 | 26.5 | 20.1 |
| 42213 | 30.8 | 26.6 | 24.0 |
| 42214 | 32.1 | 26.5 | 22.8 |
| 42215 | 30.0 | 25.8 | 23.2 |
| 42216 | 33.0 | 27.3 | 23.1 |
| 42217 | 33.300000000000004 | 27.7 | 22.2 |
| 42218 | 32.300000000000004 | 26.3 | 21.7 |
| 42219 | 32.800000000000004 | 26.6 | 22.9 |
| 42220 | 34.1 | 27.5 | 22.9 |
| 42221 | 35.800000000000004 | 28.3 | 22.9 |
| 42222 | 35.800000000000004 | 29.0 | 22.8 |
| 42223 | 34.5 | 27.3 | 21.8 |
| 42224 | 27.4 | 24.0 | 20.2 |
| 42225 | 27.9 | 23.8 | 20.5 |
| 42226 | 29.7 | 26.0 | 23.0 |
| 42227 | 29.0 | 25.4 | 22.5 |
| 42228 | 28.9 | 24.3 | 21.4 |
| 42229 | 23.6 | 22.2 | 21.0 |
| 42230 | 25.4 | 22.4 | 20.8 |
| 42231 | 29.7 | 24.3 | 20.5 |
| 42232 | 27.9 | 24.2 | 21.1 |
| 42233 | 25.6 | 23.6 | 21.7 |
| 42234 | 31.3 | 25.2 | 21.1 |
| 42235 | 27.8 | 23.6 | 17.9 |
| 42236 | 26.2 | 21.2 | 16.9 |
| 42237 | 25.2 | 22.5 | 20.4 |
| 42238 | 27.9 | 23.2 | 21.6 |
| 42239 | 22.6 | 21.2 | 19.0 |
| 42240 | 23.4 | 19.7 | 15.2 |
| 42241 | 21.5 | 18.0 | 15.2 |
| 42242 | 21.7 | 18.7 | 16.5 |
| 42243 | 20.7 | 18.8 | 17.3 |
| 42244 | 23.4 | 20.1 | 17.8 |
| 42245 | 19.6 | 18.9 | 18.5 |
| 42246 | 20.1 | 18.9 | 18.1 |
| 42247 | 21.2 | 19.6 | 18.3 |
| 42248 | 24.7 | 21.4 | 19.2 |
| 42249 | 31.6 | 24.4 | 20.4 |
| 42250 | 26.3 | 22.1 | 17.5 |
| 42251 | 27.7 | 22.1 | 18.5 |
| 42252 | 26.8 | 21.3 | 15.9 |
| 42253 | 22.5 | 19.9 | 18.5 |
| 42254 | 21.9 | 19.4 | 18.5 |
| 42255 | 19.7 | 18.4 | 17.6 |
| 42256 | 22.6 | 20.4 | 17.1 |
| 42257 | 23.7 | 22.2 | 21.6 |
| 42258 | 24.5 | 21.1 | 17.6 |
| 42259 | 26.0 | 20.6 | 14.6 |
| 42260 | 25.5 | 20.4 | 16.5 |
| 42261 | 24.2 | 18.3 | 14.1 |
| 42262 | 24.0 | 17.5 | 12.0 |
| 42263 | 23.8 | 17.8 | 12.9 |
| 42264 | 22.5 | 17.3 | 13.8 |
| 42265 | 21.5 | 18.1 | 14.4 |
| 42266 | 26.9 | 20.5 | 15.7 |
| 42267 | 25.7 | 20.4 | 15.2 |
| 42268 | 25.0 | 18.0 | 12.1 |
| 42269 | 26.4 | 19.0 | 11.8 |
| 42270 | 24.9 | 17.3 | 11.3 |
| 42271 | 22.4 | 17.1 | 12.1 |
| 42272 | 19.0 | 17.1 | 15.7 |
| 42273 | 20.4 | 17.7 | 15.7 |
| 42274 | 26.1 | 19.6 | 14.3 |
| 42275 | 25.4 | 18.4 | 10.5 |
| 42276 | 20.9 | 16.9 | 10.7 |
| 42277 | 21.4 | 15.8 | 8.8 |2015
### Chart
| Category | 最高気温 | 平均気温 | |
|---|---|---|---|
| 42514 | 25.1 | 18.7 | 12.6 |
| 42515 | 22.1 | 18.6 | 16.4 |
| 42516 | 24.5 | 19.8 | 16.9 |
| 42517 | 23.5 | 18.8 | 14.4 |
| 42518 | 23.6 | 17.6 | 12.7 |
| 42519 | 22.8 | 18.4 | 12.9 |
| 42520 | 23.9 | 19.4 | 16.2 |
| 42521 | 22.2 | 18.4 | 15.2 |
| 42522 | 20.5 | 16.3 | 12.7 |
| 42523 | 18.8 | 14.8 | 11.2 |
| 42524 | 21.8 | 15.6 | 9.9 |
| 42525 | 24.0 | 18.3 | 10.1 |
| 42526 | 25.6 | 18.1 | 13.6 |
| 42527 | 21.1 | 16.6 | 13.3 |
| 42528 | 20.7 | 17.5 | 13.2 |
| 42529 | 22.5 | 19.3 | 16.9 |
| 42530 | 21.7 | 19.5 | 17.1 |
| 42531 | 29.1 | 22.4 | 16.3 |
| 42532 | 27.8 | 20.1 | 12.9 |
| 42533 | 24.1 | 20.4 | 18.3 |
| 42534 | 20.4 | 18.0 | 16.8 |
| 42535 | 20.9 | 18.3 | 16.9 |
| 42536 | 19.5 | 17.8 | 16.7 |
| 42537 | 20.6 | 19.0 | 16.6 |
| 42538 | 26.5 | 21.1 | 18.1 |
| 42539 | 27.1 | 22.8 | 18.0 |
| 42540 | 23.4 | 20.3 | 16.4 |
| 42541 | 23.3 | 20.5 | 18.5 |
| 42542 | 25.2 | 20.8 | 16.9 |
| 42543 | 23.9 | 20.0 | 17.0 |
| 42544 | 18.2 | 17.3 | 16.7 |
| 42545 | 22.1 | 19.4 | 17.0 |
| 42546 | 25.8 | 21.6 | 19.4 |
| 42547 | 23.0 | 19.8 | 16.7 |
| 42548 | 26.3 | 20.4 | 15.9 |
| 42549 | 20.5 | 19.1 | 17.8 |
| 42550 | 24.9 | 20.6 | 16.6 |
| 42551 | 20.9 | 20.2 | 19.6 |
| 42552 | 28.5 | 22.5 | 18.2 |
| 42553 | 23.4 | 22.0 | 20.5 |
| 42554 | 27.8 | 23.7 | 20.6 |
| 42555 | 28.5 | 22.9 | 17.2 |
| 42556 | 20.1 | 18.1 | 16.9 |
| 42557 | 20.9 | 19.6 | 17.0 |
| 42558 | 23.6 | 19.7 | 17.3 |
| 42559 | 25.1 | 20.7 | 17.2 |
| 42560 | 24.7 | 21.7 | 20.1 |
| 42561 | 28.7 | 23.8 | 18.3 |
| 42562 | 29.2 | 23.6 | 18.1 |
| 42563 | 26.3 | 21.4 | 16.8 |
| 42564 | 25.9 | 22.2 | 20.0 |
| 42565 | 26.3 | 22.8 | 21.2 |
| 42566 | 25.6 | 21.6 | 18.9 |
| 42567 | 24.0 | 21.1 | 18.2 |
| 42568 | 24.7 | 22.7 | 21.2 |
| 42569 | 29.8 | 25.1 | 20.6 |
| 42570 | 29.4 | 24.4 | 18.7 |
| 42571 | 25.9 | 21.8 | 18.3 |
| 42572 | 23.6 | 20.4 | 18.7 |
| 42573 | 24.6 | 20.3 | 16.4 |
| 42574 | 24.3 | 20.0 | 17.3 |
| 42575 | 25.4 | 21.0 | 18.2 |
| 42576 | 26.8 | 22.6 | 19.6 |
| 42577 | 25.9 | 23.2 | 21.1 |
| 42578 | 26.5 | 23.8 | 22.3 |
| 42579 | 27.8 | 24.6 | 22.6 |
| 42580 | 29.4 | 25.6 | 22.8 |
| 42581 | 29.4 | 24.9 | 20.7 |
| 42582 | 29.8 | 25.8 | 22.2 |
| 42583 | 30.7 | 26.0 | 23.4 |
| 42584 | 29.7 | 25.0 | 23.0 |
| 42585 | 29.2 | 25.3 | 22.9 |
| 42586 | 32.6 | 26.5 | 21.9 |
| 42587 | 33.4 | 26.8 | 21.6 |
| 42588 | 31.9 | 26.4 | 21.7 |
| 42589 | 30.4 | 25.8 | 21.0 |
| 42590 | 31.0 | 25.6 | 21.3 |
| 42591 | 32.1 | 26.1 | 20.6 |
| 42592 | 29.1 | 23.8 | 18.6 |
| 42593 | 28.3 | 23.3 | 19.6 |
| 42594 | 28.1 | 22.4 | 18.4 |
| 42595 | 26.9 | 21.4 | 16.5 |
| 42596 | 27.4 | 21.5 | 16.3 |
| 42597 | 27.5 | 23.1 | 18.0 |
| 42598 | 29.8 | 26.6 | 24.0 |
| 42599 | 30.3 | 25.8 | 24.1 |
| 42600 | 26.7 | 24.5 | 23.1 |
| 42601 | 29.3 | 26.1 | 23.8 |
| 42602 | 29.1 | 25.8 | 23.5 |
| 42603 | 31.9 | 27.0 | 23.0 |
| 42604 | 29.1 | 26.2 | 23.4 |
| 42605 | 30.9 | 24.2 | 21.0 |
| 42606 | 28.2 | 23.9 | 20.5 |
| 42607 | 28.0 | 25.4 | 23.2 |
| 42608 | 29.0 | 25.3 | 22.0 |
| 42609 | 22.1 | 20.2 | 18.4 |
| 42610 | 24.5 | 20.9 | 18.1 |
| 42611 | 27.4 | 24.2 | 20.7 |
| 42612 | 27.3 | 24.0 | 21.1 |
| 42613 | 27.5 | 23.5 | 19.7 |
| 42614 | 29.1 | 24.2 | 20.4 |
| 42615 | 27.9 | 23.1 | 18.8 |
| 42616 | 27.7 | 23.6 | 20.2 |
| 42617 | 28.7 | 24.6 | 21.9 |
| 42618 | 29.8 | 26.0 | 23.7 |
| 42619 | 30.3 | 26.4 | 22.6 |
| 42620 | 28.4 | 23.3 | 18.7 |
| 42621 | 24.9 | 21.8 | 18.8 |
| 42622 | 29.1 | 23.4 | 17.1 |
| 42623 | 28.9 | 21.9 | 15.2 |
| 42624 | 22.2 | 19.9 | 17.2 |
| 42625 | 22.5 | 20.4 | 18.8 |
| 42626 | 20.3 | 19.3 | 18.3 |
| 42627 | 25.6 | 21.2 | 18.1 |
| 42628 | 27.8 | 21.7 | 18.0 |
| 42629 | 24.8 | 21.5 | 19.3 |
| 42630 | 24.6 | 21.0 | 17.9 |
| 42631 | 19.6 | 18.9 | 18.5 |
| 42632 | 19.8 | 18.5 | 17.7 |
| 42633 | 18.5 | 16.7 | 14.7 |
| 42634 | 22.7 | 17.8 | 14.3 |
| 42635 | 22.3 | 18.1 | 16.3 |
| 42636 | 24.2 | 19.4 | 16.5 |
| 42637 | 25.3 | 20.5 | 16.8 |
| 42638 | 25.3 | 21.1 | 18.9 |
| 42639 | 24.8 | 21.3 | 19.2 |
| 42640 | 24.9 | 21.7 | 19.2 |
| 42641 | 24.5 | 22.5 | 20.7 |
| 42642 | 21.9 | 18.4 | 12.5 |
| 42643 | 21.7 | 15.9 | 9.8 |2016
Figure S1. Daily maximum, minimum and average air temperature during the experiments.

## Slide 2
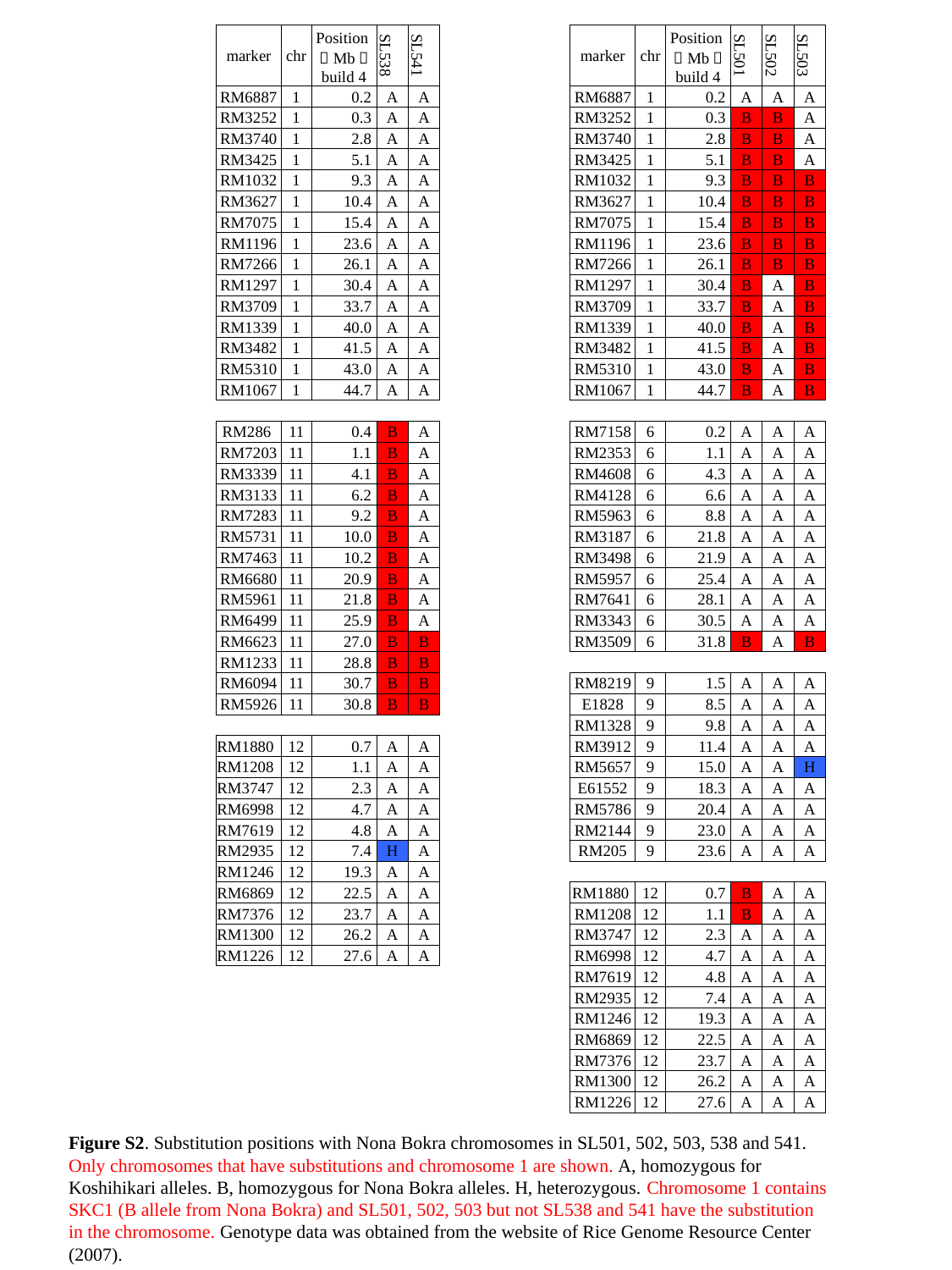

| marker | chr | Position（Mb） build 4 | SL538 | SL541 | | | marker | chr | Position（Mb） build 4 | SL501 | SL502 | SL503 |
| --- | --- | --- | --- | --- | --- | --- | --- | --- | --- | --- | --- | --- |
| RM6887 | 1 | 0.2 | A | A | | | RM6887 | 1 | 0.2 | A | A | A |
| RM3252 | 1 | 0.3 | A | A | | | RM3252 | 1 | 0.3 | B | B | A |
| RM3740 | 1 | 2.8 | A | A | | | RM3740 | 1 | 2.8 | B | B | A |
| RM3425 | 1 | 5.1 | A | A | | | RM3425 | 1 | 5.1 | B | B | A |
| RM1032 | 1 | 9.3 | A | A | | | RM1032 | 1 | 9.3 | B | B | B |
| RM3627 | 1 | 10.4 | A | A | | | RM3627 | 1 | 10.4 | B | B | B |
| RM7075 | 1 | 15.4 | A | A | | | RM7075 | 1 | 15.4 | B | B | B |
| RM1196 | 1 | 23.6 | A | A | | | RM1196 | 1 | 23.6 | B | B | B |
| RM7266 | 1 | 26.1 | A | A | | | RM7266 | 1 | 26.1 | B | B | B |
| RM1297 | 1 | 30.4 | A | A | | | RM1297 | 1 | 30.4 | B | A | B |
| RM3709 | 1 | 33.7 | A | A | | | RM3709 | 1 | 33.7 | B | A | B |
| RM1339 | 1 | 40.0 | A | A | | | RM1339 | 1 | 40.0 | B | A | B |
| RM3482 | 1 | 41.5 | A | A | | | RM3482 | 1 | 41.5 | B | A | B |
| RM5310 | 1 | 43.0 | A | A | | | RM5310 | 1 | 43.0 | B | A | B |
| RM1067 | 1 | 44.7 | A | A | | | RM1067 | 1 | 44.7 | B | A | B |
| | | | | | | | | | | | | |
| RM286 | 11 | 0.4 | B | A | | | RM7158 | 6 | 0.2 | A | A | A |
| RM7203 | 11 | 1.1 | B | A | | | RM2353 | 6 | 1.1 | A | A | A |
| RM3339 | 11 | 4.1 | B | A | | | RM4608 | 6 | 4.3 | A | A | A |
| RM3133 | 11 | 6.2 | B | A | | | RM4128 | 6 | 6.6 | A | A | A |
| RM7283 | 11 | 9.2 | B | A | | | RM5963 | 6 | 8.8 | A | A | A |
| RM5731 | 11 | 10.0 | B | A | | | RM3187 | 6 | 21.8 | A | A | A |
| RM7463 | 11 | 10.2 | B | A | | | RM3498 | 6 | 21.9 | A | A | A |
| RM6680 | 11 | 20.9 | B | A | | | RM5957 | 6 | 25.4 | A | A | A |
| RM5961 | 11 | 21.8 | B | A | | | RM7641 | 6 | 28.1 | A | A | A |
| RM6499 | 11 | 25.9 | B | A | | | RM3343 | 6 | 30.5 | A | A | A |
| RM6623 | 11 | 27.0 | B | B | | | RM3509 | 6 | 31.8 | B | A | B |
| RM1233 | 11 | 28.8 | B | B | | | | | | | | |
| RM6094 | 11 | 30.7 | B | B | | | RM8219 | 9 | 1.5 | A | A | A |
| RM5926 | 11 | 30.8 | B | B | | | E1828 | 9 | 8.5 | A | A | A |
| | | | | | | | RM1328 | 9 | 9.8 | A | A | A |
| RM1880 | 12 | 0.7 | A | A | | | RM3912 | 9 | 11.4 | A | A | A |
| RM1208 | 12 | 1.1 | A | A | | | RM5657 | 9 | 15.0 | A | A | H |
| RM3747 | 12 | 2.3 | A | A | | | E61552 | 9 | 18.3 | A | A | A |
| RM6998 | 12 | 4.7 | A | A | | | RM5786 | 9 | 20.4 | A | A | A |
| RM7619 | 12 | 4.8 | A | A | | | RM2144 | 9 | 23.0 | A | A | A |
| RM2935 | 12 | 7.4 | H | A | | | RM205 | 9 | 23.6 | A | A | A |
| RM1246 | 12 | 19.3 | A | A | | | | | | | | |
| RM6869 | 12 | 22.5 | A | A | | | RM1880 | 12 | 0.7 | B | A | A |
| RM7376 | 12 | 23.7 | A | A | | | RM1208 | 12 | 1.1 | B | A | A |
| RM1300 | 12 | 26.2 | A | A | | | RM3747 | 12 | 2.3 | A | A | A |
| RM1226 | 12 | 27.6 | A | A | | | RM6998 | 12 | 4.7 | A | A | A |
| | | | | | | | RM7619 | 12 | 4.8 | A | A | A |
| | | | | | | | RM2935 | 12 | 7.4 | A | A | A |
| | | | | | | | RM1246 | 12 | 19.3 | A | A | A |
| | | | | | | | RM6869 | 12 | 22.5 | A | A | A |
| | | | | | | | RM7376 | 12 | 23.7 | A | A | A |
| | | | | | | | RM1300 | 12 | 26.2 | A | A | A |
| | | | | | | | RM1226 | 12 | 27.6 | A | A | A |
Figure S2. Substitution positions with Nona Bokra chromosomes in SL501, 502, 503, 538 and 541.
Only chromosomes that have substitutions and chromosome 1 are shown. A, homozygous for Koshihikari alleles. B, homozygous for Nona Bokra alleles. H, heterozygous. Chromosome 1 contains SKC1 (B allele from Nona Bokra) and SL501, 502, 503 but not SL538 and 541 have the substitution in the chromosome. Genotype data was obtained from the website of Rice Genome Resource Center (2007).

## Slide 3
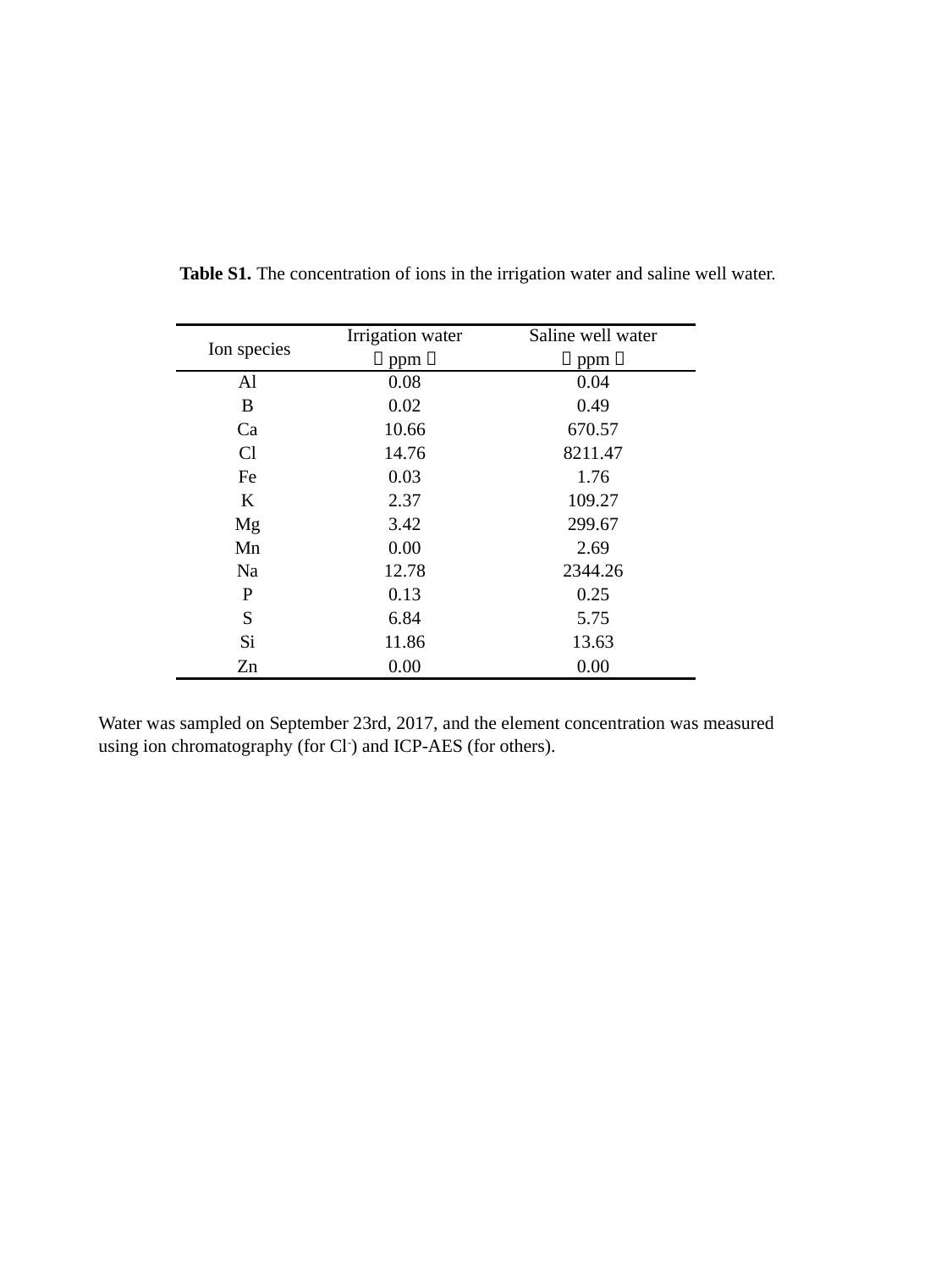

Table S1. The concentration of ions in the irrigation water and saline well water.
| Ion species | Irrigation water （ppm） | Saline well water （ppm） |
| --- | --- | --- |
| Al | 0.08 | 0.04 |
| B | 0.02 | 0.49 |
| Ca | 10.66 | 670.57 |
| Cl | 14.76 | 8211.47 |
| Fe | 0.03 | 1.76 |
| K | 2.37 | 109.27 |
| Mg | 3.42 | 299.67 |
| Mn | 0.00 | 2.69 |
| Na | 12.78 | 2344.26 |
| P | 0.13 | 0.25 |
| S | 6.84 | 5.75 |
| Si | 11.86 | 13.63 |
| Zn | 0.00 | 0.00 |
Water was sampled on September 23rd, 2017, and the element concentration was measured using ion chromatography (for Cl-) and ICP-AES (for others).

## Slide 4
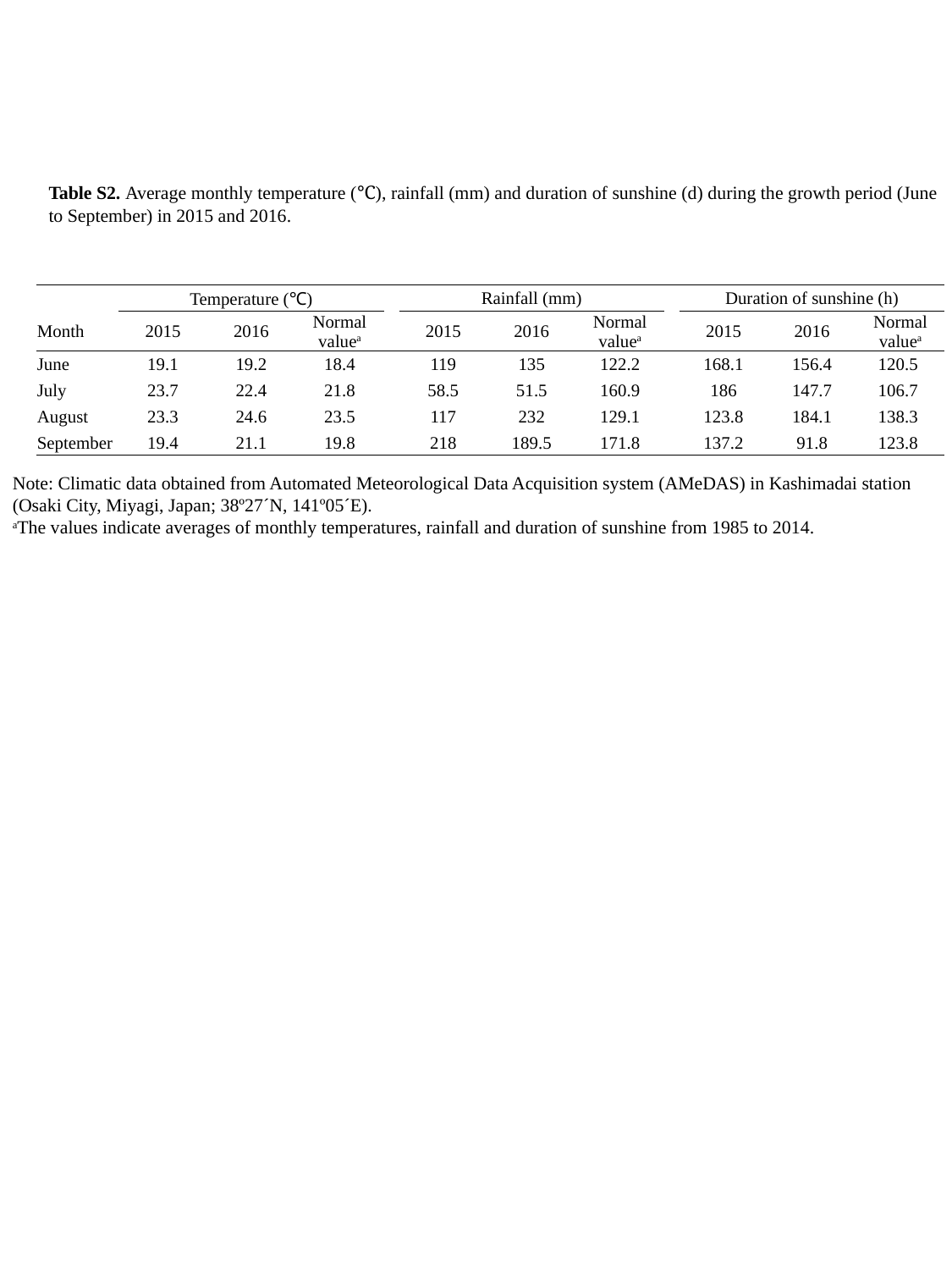

Table S2. Average monthly temperature (℃), rainfall (mm) and duration of sunshine (d) during the growth period (June to September) in 2015 and 2016.
| | Temperature (℃) | | | | Rainfall (mm) | | | | Duration of sunshine (h) | | |
| --- | --- | --- | --- | --- | --- | --- | --- | --- | --- | --- | --- |
| Month | 2015 | 2016 | Normal valuea | | 2015 | 2016 | Normal valuea | | 2015 | 2016 | Normal valuea |
| June | 19.1 | 19.2 | 18.4 | | 119 | 135 | 122.2 | | 168.1 | 156.4 | 120.5 |
| July | 23.7 | 22.4 | 21.8 | | 58.5 | 51.5 | 160.9 | | 186 | 147.7 | 106.7 |
| August | 23.3 | 24.6 | 23.5 | | 117 | 232 | 129.1 | | 123.8 | 184.1 | 138.3 |
| September | 19.4 | 21.1 | 19.8 | | 218 | 189.5 | 171.8 | | 137.2 | 91.8 | 123.8 |
Note: Climatic data obtained from Automated Meteorological Data Acquisition system (AMeDAS) in Kashimadai station (Osaki City, Miyagi, Japan; 38º27´N, 141º05´E).
aThe values indicate averages of monthly temperatures, rainfall and duration of sunshine from 1985 to 2014.

## Slide 5
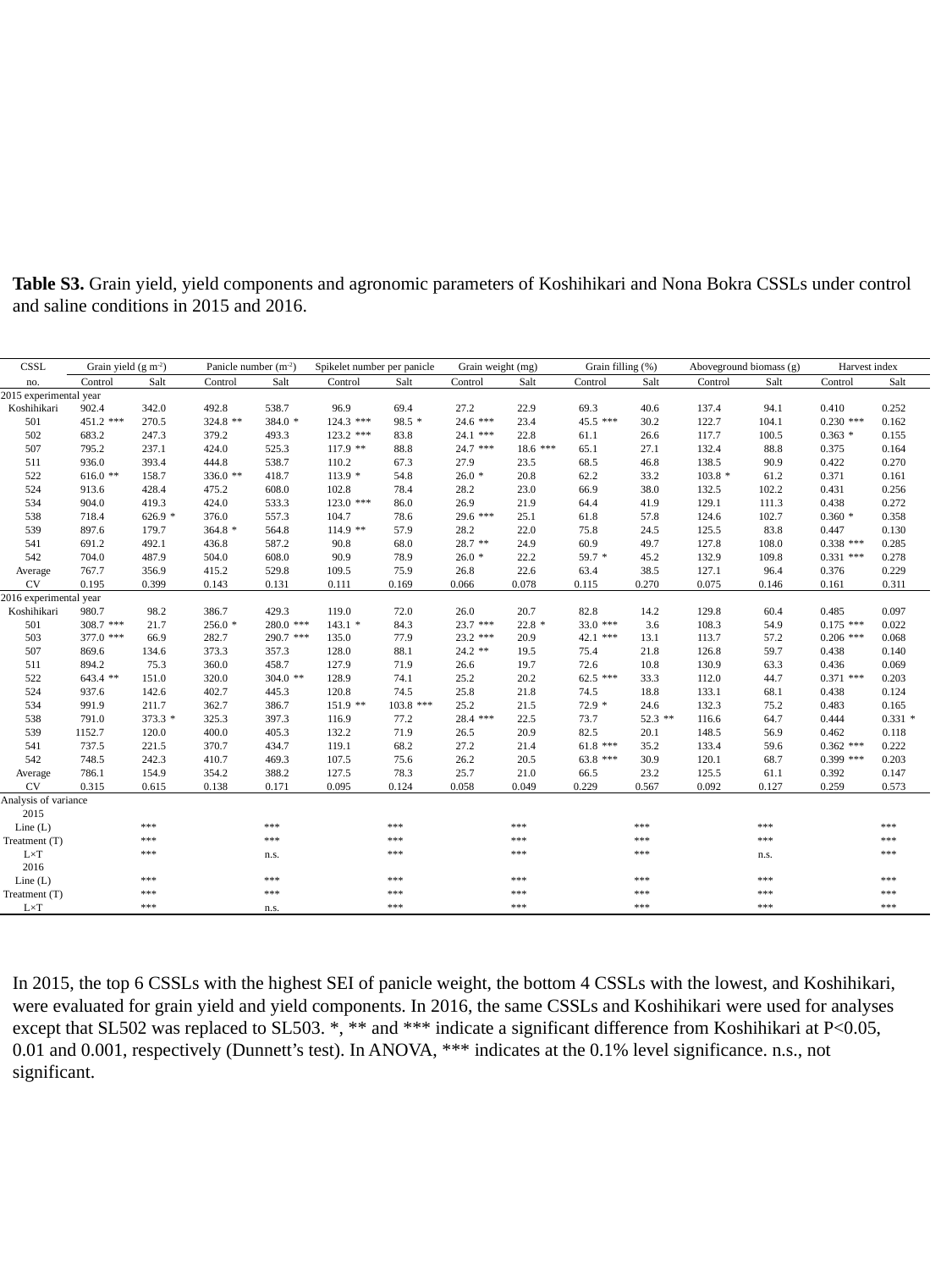

Table S3. Grain yield, yield components and agronomic parameters of Koshihikari and Nona Bokra CSSLs under control and saline conditions in 2015 and 2016.
| | | | | | | | | | | | | | | | | | | | | | | | | | | | | |
| --- | --- | --- | --- | --- | --- | --- | --- | --- | --- | --- | --- | --- | --- | --- | --- | --- | --- | --- | --- | --- | --- | --- | --- | --- | --- | --- | --- | --- |
| CSSL | Grain yield (g m-2) | | | | Panicle number (m-2) | | | | Spikelet number per panicle | | | | Grain weight (mg) | | | | Grain filling (%) | | | | Aboveground biomass (g) | | | | Harvest index | | | |
| no. | Control | | Salt | | Control | | Salt | | Control | | Salt | | Control | | Salt | | Control | | Salt | | Control | | Salt | | Control | | Salt | |
| 2015 experimental year | | | | | | | | | | | | | | | | | | | | | | | | | | | | |
| Koshihikari | 902.4 | | 342.0 | | 492.8 | | 538.7 | | 96.9 | | 69.4 | | 27.2 | | 22.9 | | 69.3 | | 40.6 | | 137.4 | | 94.1 | | 0.410 | | 0.252 | |
| 501 | 451.2 | \*\*\* | 270.5 | | 324.8 | \*\* | 384.0 | \* | 124.3 | \*\*\* | 98.5 | \* | 24.6 | \*\*\* | 23.4 | | 45.5 | \*\*\* | 30.2 | | 122.7 | | 104.1 | | 0.230 | \*\*\* | 0.162 | |
| 502 | 683.2 | | 247.3 | | 379.2 | | 493.3 | | 123.2 | \*\*\* | 83.8 | | 24.1 | \*\*\* | 22.8 | | 61.1 | | 26.6 | | 117.7 | | 100.5 | | 0.363 | \* | 0.155 | |
| 507 | 795.2 | | 237.1 | | 424.0 | | 525.3 | | 117.9 | \*\* | 88.8 | | 24.7 | \*\*\* | 18.6 | \*\*\* | 65.1 | | 27.1 | | 132.4 | | 88.8 | | 0.375 | | 0.164 | |
| 511 | 936.0 | | 393.4 | | 444.8 | | 538.7 | | 110.2 | | 67.3 | | 27.9 | | 23.5 | | 68.5 | | 46.8 | | 138.5 | | 90.9 | | 0.422 | | 0.270 | |
| 522 | 616.0 | \*\* | 158.7 | | 336.0 | \*\* | 418.7 | | 113.9 | \* | 54.8 | | 26.0 | \* | 20.8 | | 62.2 | | 33.2 | | 103.8 | \* | 61.2 | | 0.371 | | 0.161 | |
| 524 | 913.6 | | 428.4 | | 475.2 | | 608.0 | | 102.8 | | 78.4 | | 28.2 | | 23.0 | | 66.9 | | 38.0 | | 132.5 | | 102.2 | | 0.431 | | 0.256 | |
| 534 | 904.0 | | 419.3 | | 424.0 | | 533.3 | | 123.0 | \*\*\* | 86.0 | | 26.9 | | 21.9 | | 64.4 | | 41.9 | | 129.1 | | 111.3 | | 0.438 | | 0.272 | |
| 538 | 718.4 | | 626.9 | \* | 376.0 | | 557.3 | | 104.7 | | 78.6 | | 29.6 | \*\*\* | 25.1 | | 61.8 | | 57.8 | | 124.6 | | 102.7 | | 0.360 | \* | 0.358 | |
| 539 | 897.6 | | 179.7 | | 364.8 | \* | 564.8 | | 114.9 | \*\* | 57.9 | | 28.2 | | 22.0 | | 75.8 | | 24.5 | | 125.5 | | 83.8 | | 0.447 | | 0.130 | |
| 541 | 691.2 | | 492.1 | | 436.8 | | 587.2 | | 90.8 | | 68.0 | | 28.7 | \*\* | 24.9 | | 60.9 | | 49.7 | | 127.8 | | 108.0 | | 0.338 | \*\*\* | 0.285 | |
| 542 | 704.0 | | 487.9 | | 504.0 | | 608.0 | | 90.9 | | 78.9 | | 26.0 | \* | 22.2 | | 59.7 | \* | 45.2 | | 132.9 | | 109.8 | | 0.331 | \*\*\* | 0.278 | |
| Average | 767.7 | | 356.9 | | 415.2 | | 529.8 | | 109.5 | | 75.9 | | 26.8 | | 22.6 | | 63.4 | | 38.5 | | 127.1 | | 96.4 | | 0.376 | | 0.229 | |
| CV | 0.195 | | 0.399 | | 0.143 | | 0.131 | | 0.111 | | 0.169 | | 0.066 | | 0.078 | | 0.115 | | 0.270 | | 0.075 | | 0.146 | | 0.161 | | 0.311 | |
| 2016 experimental year | | | | | | | | | | | | | | | | | | | | | | | | | | | | |
| Koshihikari | 980.7 | | 98.2 | | 386.7 | | 429.3 | | 119.0 | | 72.0 | | 26.0 | | 20.7 | | 82.8 | | 14.2 | | 129.8 | | 60.4 | | 0.485 | | 0.097 | |
| 501 | 308.7 | \*\*\* | 21.7 | | 256.0 | \* | 280.0 | \*\*\* | 143.1 | \* | 84.3 | | 23.7 | \*\*\* | 22.8 | \* | 33.0 | \*\*\* | 3.6 | | 108.3 | | 54.9 | | 0.175 | \*\*\* | 0.022 | |
| 503 | 377.0 | \*\*\* | 66.9 | | 282.7 | | 290.7 | \*\*\* | 135.0 | | 77.9 | | 23.2 | \*\*\* | 20.9 | | 42.1 | \*\*\* | 13.1 | | 113.7 | | 57.2 | | 0.206 | \*\*\* | 0.068 | |
| 507 | 869.6 | | 134.6 | | 373.3 | | 357.3 | | 128.0 | | 88.1 | | 24.2 | \*\* | 19.5 | | 75.4 | | 21.8 | | 126.8 | | 59.7 | | 0.438 | | 0.140 | |
| 511 | 894.2 | | 75.3 | | 360.0 | | 458.7 | | 127.9 | | 71.9 | | 26.6 | | 19.7 | | 72.6 | | 10.8 | | 130.9 | | 63.3 | | 0.436 | | 0.069 | |
| 522 | 643.4 | \*\* | 151.0 | | 320.0 | | 304.0 | \*\* | 128.9 | | 74.1 | | 25.2 | | 20.2 | | 62.5 | \*\*\* | 33.3 | | 112.0 | | 44.7 | | 0.371 | \*\*\* | 0.203 | |
| 524 | 937.6 | | 142.6 | | 402.7 | | 445.3 | | 120.8 | | 74.5 | | 25.8 | | 21.8 | | 74.5 | | 18.8 | | 133.1 | | 68.1 | | 0.438 | | 0.124 | |
| 534 | 991.9 | | 211.7 | | 362.7 | | 386.7 | | 151.9 | \*\* | 103.8 | \*\*\* | 25.2 | | 21.5 | | 72.9 | \* | 24.6 | | 132.3 | | 75.2 | | 0.483 | | 0.165 | |
| 538 | 791.0 | | 373.3 | \* | 325.3 | | 397.3 | | 116.9 | | 77.2 | | 28.4 | \*\*\* | 22.5 | | 73.7 | | 52.3 | \*\* | 116.6 | | 64.7 | | 0.444 | | 0.331 | \* |
| 539 | 1152.7 | | 120.0 | | 400.0 | | 405.3 | | 132.2 | | 71.9 | | 26.5 | | 20.9 | | 82.5 | | 20.1 | | 148.5 | | 56.9 | | 0.462 | | 0.118 | |
| 541 | 737.5 | | 221.5 | | 370.7 | | 434.7 | | 119.1 | | 68.2 | | 27.2 | | 21.4 | | 61.8 | \*\*\* | 35.2 | | 133.4 | | 59.6 | | 0.362 | \*\*\* | 0.222 | |
| 542 | 748.5 | | 242.3 | | 410.7 | | 469.3 | | 107.5 | | 75.6 | | 26.2 | | 20.5 | | 63.8 | \*\*\* | 30.9 | | 120.1 | | 68.7 | | 0.399 | \*\*\* | 0.203 | |
| Average | 786.1 | | 154.9 | | 354.2 | | 388.2 | | 127.5 | | 78.3 | | 25.7 | | 21.0 | | 66.5 | | 23.2 | | 125.5 | | 61.1 | | 0.392 | | 0.147 | |
| CV | 0.315 | | 0.615 | | 0.138 | | 0.171 | | 0.095 | | 0.124 | | 0.058 | | 0.049 | | 0.229 | | 0.567 | | 0.092 | | 0.127 | | 0.259 | | 0.573 | |
| Analysis of variance | | | | | | | | | | | | | | | | | | | | | | | | | | | | |
| 2015 | | | | | | | | | | | | | | | | | | | | | | | | | | | | |
| Line (L) | | | \*\*\* | | | | \*\*\* | | | | \*\*\* | | | | \*\*\* | | | | \*\*\* | | | | \*\*\* | | | | \*\*\* | |
| Treatment (T) | | | \*\*\* | | | | \*\*\* | | | | \*\*\* | | | | \*\*\* | | | | \*\*\* | | | | \*\*\* | | | | \*\*\* | |
| L×T | | | \*\*\* | | | | n.s. | | | | \*\*\* | | | | \*\*\* | | | | \*\*\* | | | | n.s. | | | | \*\*\* | |
| 2016 | | | | | | | | | | | | | | | | | | | | | | | | | | | | |
| Line (L) | | | \*\*\* | | | | \*\*\* | | | | \*\*\* | | | | \*\*\* | | | | \*\*\* | | | | \*\*\* | | | | \*\*\* | |
| Treatment (T) | | | \*\*\* | | | | \*\*\* | | | | \*\*\* | | | | \*\*\* | | | | \*\*\* | | | | \*\*\* | | | | \*\*\* | |
| L×T | | | \*\*\* | | | | n.s. | | | | \*\*\* | | | | \*\*\* | | | | \*\*\* | | | | \*\*\* | | | | \*\*\* | |
| | | | | | | | | | | | | | | | | | | | | | | | | | | | | |
In 2015, the top 6 CSSLs with the highest SEI of panicle weight, the bottom 4 CSSLs with the lowest, and Koshihikari, were evaluated for grain yield and yield components. In 2016, the same CSSLs and Koshihikari were used for analyses except that SL502 was replaced to SL503. *, ** and *** indicate a significant difference from Koshihikari at P<0.05, 0.01 and 0.001, respectively (Dunnett’s test). In ANOVA, *** indicates at the 0.1% level significance. n.s., not significant.
